# Effects of *Funneliformis mosseae* on Root Metabolites and Rhizosphere Soil Properties to Continuously-Cropped Soybean in the Potted-Experiments

**DOI:** 10.3390/ijms19082160

**Published:** 2018-07-24

**Authors:** Jia-Qi Cui, Hai-Bing Sun, Ming-Bo Sun, Rui-Ting Liang, Wei-Guang Jie, Bai-Yan Cai

**Affiliations:** 1 Engineering Research Center of Agricultural Microbiology Technology, Ministry of Education, Heilongjiang University, Harbin 150500, China; cuijiaqi2015@126.com (J.-Q.C.); sunhaibing126@126.com (H.-B.S.); sunmb.lpec@sinopec.com (M.-B.S.); tingwaiwaibaobei@163.com (R.-T.L.); jieweiguang2007@126.com (W.-G.J.); 2 Heilongjiang Provincial Key Laboratory of Ecological Restoration and Resource Utilization for Cold Region, School of Life Sciences, Heilongjiang University, Harbin 150080, China; 3 Key Laboratory of Molecular Biology, College of Heilongjiang Province, School of Life Sciences, Heilongjiang University, Harbin 150080, China

**Keywords:** potted-soybean, *Funneliformis mosseae*, root metabolites, biomass, soil properties

## Abstract

Continuous cropping in soybean is increasingly practiced in Heilongjiang Province, leading to substantial yield reductions and quality degradation. Arbuscular mycorrhizal fungi (AMF) are soil microorganisms that form mutualistic interactions with plant roots and can restore the plant rhizosphere microenvironment. In this study, two soybean lines (HN48 and HN66) were chosen as experimental materials, which were planted in different years of continuous cropping soybean soils and were inoculated or not with *Funneliformis mosseae* in potted-experiments. Ultimately, analysis of root tissue metabolome and root exudates, soil physicochemical properties, plant biomass, as well as rhizosphere soil properties in different experimental treatments, inoculated or not with *F. mosseae*, was performed. Experimental results showed that: (a) The disease index of soybean root rot was significantly lower in the treatment group than in the control group, and there were differences in disease index and the resistance effect of *F. mosseae* between the two cultivars; (b) compared with the control, the root tissue metabolome and root exudates remained unchanged, but there were changes in the relative amounts in the treatment group, and the abundant metabolites differed by soybean cultivar; (c) soybean biomass was significantly higher in the treatment group than in the control group, and the effect of *F. mosseae* on biomass differed with respect to the soybean cultivar; and (d) there were differences in the physiochemical indexes of soybean rhizosphere soil between the treatment and control groups, and the repairing effect of *F. mosseae* differed between the two cultivars. Therefore, *F. mosseae* can increase the biomass of continuously cropped soybean, improve the physicochemical properties of the rhizosphere soil, regulate the root metabolite profiles, and alleviate barriers to continuous cropping in potted-experiments of soybean.

## 1. Introduction

Soil type, crop type, and planting practice are key factors that impact crop yield and quality in agricultural production [1,2]. In recent years, changes to the soybean planting practices in Heilongjiang Province, namely long-term continuous cropping, have accumulated organic acids in the rhizosphere of soybean and caused the soil pH to decrease (changing the soil from neutral to acidic). Acidic soil benefits the growth of fungi (*Penicillium* sp., *Fusarium* sp., and *Rhizopus* sp.) and inhibits the reproduction of bacteria and actinomycetes [3,4]. Meanwhile, long-term continuous cropping would lead the soil enzyme and organic matter content to decline. Therefore, the soybean field soils have gradually been transformed from “bacterial type” high-fertility soils to “fungal type” low-fertility soils [2], which has led to a significantly higher disease index of soybean root rot. This in turn has had a significant negative effect on crop yield, mainly due to mixed infections by the soil inhabiting fungus *Fusarium oxysporum* and the oomycetes *Phytophthora* sp. and *Pythium* sp. [5,6]. *Phytophthora sojae*, *Pythium* sp., *Fusarium* sp., and *Rhizoctonia solani* are dominant soybean root rot fungi. Long-term continuous cropping in soybean causes substantial changes to the type and number of microbial communities in soil [1,7], enrichment of poisonous and harmful secondary metabolites secreted by plants [8,9], and changes in soil enzyme activity [10,11], which eventually lead to the frequent occurrence of soybean diseases and pests, followed by significant reductions in soybean yield and quality.

*Glycine max* [L.] Merr. is an important oil crop in China, providing abundant lipid and protein resources for the human diet [12]. Therefore, researchers are working on applying efficient, economical, and environmentally friendly bioremediation technology (plant–microbial symbionts) for the prevention and control of crop diseases and pests. Arbuscular mycorrhizal fungi (AMF) are obligate mutualistic symbionts that form specific interactions with plant roots in the soil microecosystem [13,14]. AMF establish a symbiotic relationship with terrestrial tracheophyte root systems to promote the absorption of water and minerals (mainly P, but also N, K, Zn, and Mn) [14,15], accelerate the formation of chloroplasts, and improve the photosynthesis rate and plant biomass [16,17]. AMF can play a role in the regulation of the plant defense response through its effect on plant metabolism and the expression of defense-related genes [18,19], improving resistance to diseases, pests, drought [20], and heavy metal pollution [21,22], as well as in maintaining soil ecosystem diversity and microecosystem stability.

Metabolomics has been widely applied to qualitative and quantitative studies of metabolites in diverse test samples. The non-target or complete metabolome aims to comprehensively describe all potential metabolites in one tissue without bias. At the same time, GC-MS (Gas Chromatography–Mass Spectrometry) and LC-MS (Liquid Chromatography–Mass Spectrometry) have been widely used to detecting a variety of plant metabolites [23,24], However, there are few studies reporting the results of applying metabolomics to the understanding of how AMF alleviates problems in continuous cropping. Therefore, two soybean cultivars that differ in terms of root rot resistance were selected as the experimental material in this study. We inoculated them with *Funneliformis mosseae* (T.H. Nicolson & Gerd.) [25] to investigate its effect on soybean biomass, rhizosphere soil physicochemical properties, and root metabolites in two different cultivars over three years of continuous cropping. Our objective was to explore the way in which *F. mosseae* alleviates the incidence of root rot and increases the biomass in soybeans grown in continuous cropping systems.

## 2. Results

### 2.1. Effect of F. mosseae on Root Rot Index and the AMF Colonization Rate in Different Conditions Potted-Soybean

**Figure 1:**
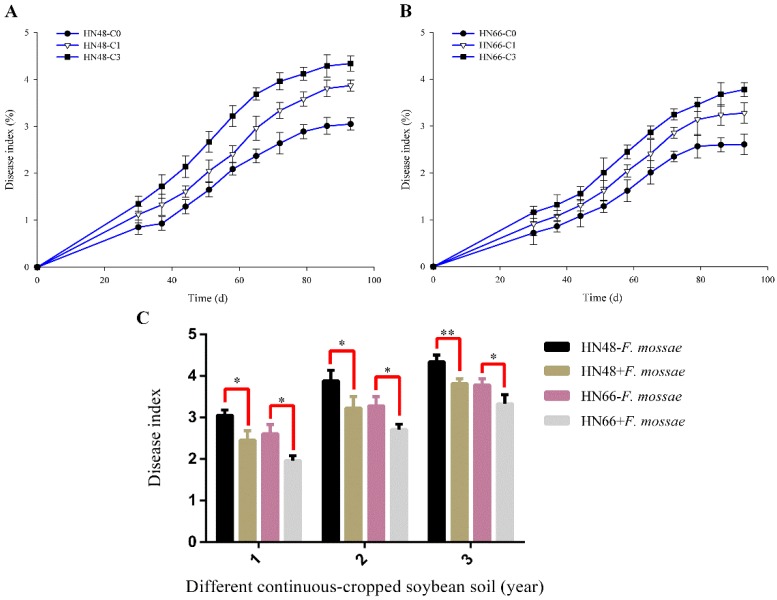
The root rot disease index under different continuous-cropped year soil in potted-experiments of soybean: 1 means soybean were planted the normal soil; 2 means soybean were planted in one-year continuous cropping soybean soil; 3 means soybean were planted in three-year continuous cropping soybean soil; C0 means soybean were planted the normal soil without inoculated *F. mosseae*; C1 means soybean were planted in one-year continuous cropping soybean soil without inoculated *F. mosseae*; and C3 means soybean were planted in three-year continuous cropping soybean soil without inoculated *F. mosseae*. Note: All experiments were conducted in potted-experiments. (**A**,**B**) *x*-axis represents which day detected the root rot disease index of two soybean cultivars; and (**C**) *x*-axis represents the year of continuous cropping soybean soil. Each value represents the average of six independent experiments and the error bars represent standard deviations. Asterisks indicate the significance of differences between the samples. *p* values were calculated by Student’s *t*-test. Single asterisk indicates *p* < 0.05; double asterisks indicate *p* < 0.01.

**Figure 2:**
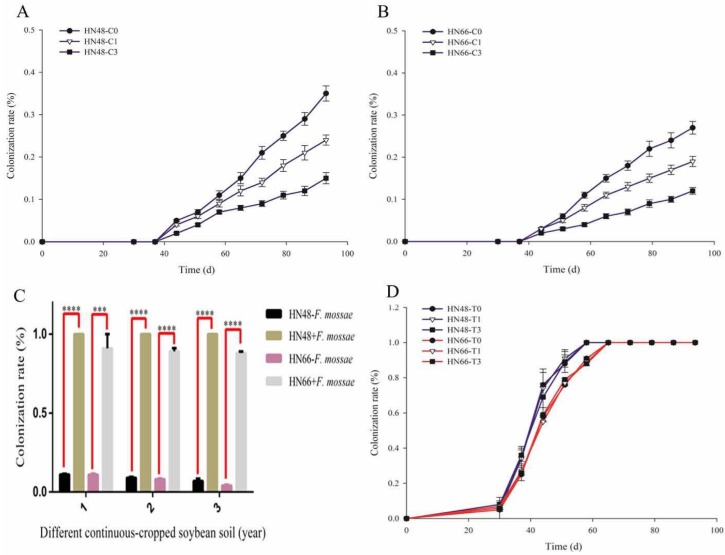
The AMF colonization rate of two soybean cultivars under different continuous-cropped year soil in potted-experiments: 1 means soybean were planted the normal soil; 2 means soybean were planted in one-year continuous cropping soybean soil; 3 means soybean were planted in three-year continuous cropping soybean soil; C0/T0 means soybean were planted the normal soil without/with inoculated *F. mosseae*; C1/T1 means soybean were planted in one-year continuous cropping soybean soil without/with inoculated *F. mosseae*; C3/T3 means soybean were planted in three-year continuous cropping soybean soil without/with inoculated *F. mosseae*. Note: All experiments were conducted in potted-experiments. (**A**,**B**,**D**) *x*-axis represents which day detected the AMF colonization rate of two soybean cultivars; and (**C**) represents the year of continuous cropping soybean soil. Each value represents the average of six independent experiments and the error bars represent standard deviations. Asterisks indicate the significance of differences between the samples. *p* values were calculated by Student’s *t*-test. Quadruple asterisk indicates *p* < 0.0001.

Morphological observation and acid fuchsin staining were used to analyze the effect of *F. mosseae* inoculation on the index of root rot (Figure 1) and the AMF colonization rate (Figure 2) in potted-soybean under different conditions.

The soybean root rot index gradually increased as the growth stage advanced, and it also increased with increasing years of continuous cropping (Figure 1A,B). After *F. mosseae* inoculation, the root rot disease index in soybean was lower in the treatment group than in the control group when the soybean had grown 93 days (Figure 1C).

The AMF colonization rate decreased with increasing years of continuous cropping (Figure 2A,B). Ninety-three days after soybean was planted in the pot, *F. mosseae* inoculation significantly increased the AMF colonization rate in continuously cropped soybean (Figure 2C). After *F. mosseae* inoculation, the AMF colonization rate in the treatment groups increased significantly, and the colonization rate reached 100% in HN48 before it did in HN66 (Figure 2D).

### 2.2. Total DNA Extraction and Specific DNA Fragment Amplification from Root and Rhizosphere Soil

The total DNA amplification results were examined on 1% agarose gels, and the bands were found in approximately 15,000 bp (Figure S1). The band of fungal 18S rDNA (V1 + V2) (Figure S2) and *F. mosseae* 18S rDNA NS31/Glol regions were found to be approximately 430 bp and 336 bp in length (Figure S3), respectively. The DNA amplification result of fungal and *F. mosseae* indicated that the pathogenic fungus and *F. mosseae* had infected the root of two cultivars in this stage.

### 2.3. Metabolite Profiling in Continuously Cropped Soybean under Potted-Experiments

#### 2.3.1. Metabolite Profiling of HN48 and HN66 Root Samples from Continuously Cropped Soybean under Potted-Experiments

The PCA (Figures S4A and S5A) showed that there were differences in the root tissue metabolites profiling from the root rot-susceptible cultivar HN48 and root rot-resistant cultivar HN66 for the three years of continuous cropping without or with *F. mosseae* inoculation. HN48/66-R0C (root tissue from HN48/66 were planted in normal soils without inoculated *F. mosseae*), HN48/66-R1C (root tissue from HN48/66 were planted in one-year continuous cropping soybean soil without inoculated *F. mossea*), HN48/66-R3C (root tissue from HN48/66 were planted in three-year continuous cropping soybean soil without inoculated *F. mossea*), HN48/66-R0T (root tissue from HN48/66 were planted in normal soils with inoculated *F. mosseae*), HN48/66-R1T (root tissue from HN48/66 were planted in one-year continuous cropping soybean soil with inoculated *F. mossea*) and HN48/66-R3T (root tissue from HN48/66 were planted in three-year continuous cropping soybean soil with inoculated *F. mossea*) were not in the same region of the plot, and there was a distance between the without or with inoculated *F. mosseae* groups, indicating that *F. mosseae* inoculation affects the profiling of the root tissue metabolites in the HN48 and HN66 under different years of continuous cropping.

We screened those components of the VIP (Variable Importance Plot) > 1 with a PLS-DA model in the root tissue metabolites from HN48 and HN66 under different years of continuous cropping. We regarded those components as biomarkers and used them to match the pathway of metabolites (Figures S4B and S5B). To verify whether the model is overfitted, we carried out 100 displacement response ranking verifications (Figures S4C and S5C). The eight and seven total chromatographic peaks were screened out in combination with VIP value (generally VIP > 1) from the root tissue metabolites of HN48 and HN66, respectively. We compared the total ion chromatograms of the chromatographic peaks with the NIST 11.5 database, and the corresponding compound types are shown in Table 1 and Table 2. The relative abundance of different metabolites from two soybean cultivars root tissue in the different continuous-cropped year soil are shown in Tables S1 and S2.

The root tissue differently abundant metabolites of HN48 and HN66 included organic acids, esters and organic hydrocarbon. The KEGG database was used for pathway annotation of the differently abundant metabolites, and the annotation results showed that propanoic acid, 2-(hydroxyl), belongs to the polyphenol degradation pathway, while hexadecanoic acid and *cis*-9-hexadecenoic acid belong to the fatty acid biosynthesis pathway. Our experimental results showed that *F. mosseae* inoculation affects the root tissue metabolites profile of HN48 and HN66

#### 2.3.2. Metabolite Profiling of HN48 and HN66 Soil Samples from Continuously Cropped Soybean under Potted-Experiments

The PCA (Figures S6A and S7A) showed that there were differences in the profiling of root exudates from HN48 and HN66 over the three years of continuous cropping without or with inoculated *F. mosseae*. HN48/66-S0C (rhizosphere soil from HN48/66 were planted in normal soils without inoculated *F. mosseae*), HN48/66-S1C (rhizosphere soil from HN48/66 were planted in one-year continuous cropping soybean soil without inoculated *F. mossea*), HN48/66-S3C (rhizosphere soil from HN48/66 were planted in three-year continuous cropping soybean soil without inoculated *F. mossea*), HN48/66-S0T (rhizosphere soil from HN48/66 were planted in normal soils with inoculated *F. mosseae*), HN48/66-S1T (rhizosphere soil from HN48/66 were planted in one-year continuous cropping soybean soil with inoculated *F. mossea*), and HN48/66-S3T (rhizosphere soil from HN48/66 were planted in three-year continuous cropping soybean soil with inoculated *F. mossea*) were not in the same regions, and there was a distance between the without or with inoculated *F. mosseae*, showing that *F. mosseae* inoculation affects the profiling of root exudates in the HN48 and HN66 under different years of continuous cropping.

We screened those components of the VIP (Variable Importance Plot) > 1 with a PLS-DA model in the root exudates from HN48 and HN66 under different years of continuous cropping. We regarded those components as biomarkers and used them to match the pathway of metabolites (Figures S6B and S7B). To verify whether the model is overfitted, we carried out 100 displacement response ranking verifications (Figures S6C and S7C); 8 and 10 chromatographic peaks were screened out in combination with VIP value (generally VIP > 1) from the root exudates of HN48 and HN66, respectively. The total ion chromatograms of the chromatographic peaks were compared to the NIST 11.5 database, and the corresponding compounds are shown in Table 3 and Table 4. Meanwhile, the relative abundance of different metabolites from two soybean cultivars root exudates under different continuous-cropped year soil in potted-experiments are shown in Tables S3 and S4.

The root exudates of differently abundant metabolites of HN48 and HN66 included esters, benzene homolog and derivate, naphthalene derivative, phenol and hydrocarbons. Experimental results showed that *F. mosseae* inoculation affects root exudates metabolites in HN48 and HN66.

### 2.4. Determination of Soybean Biomass and Rhizosphere Soil Physicochemical Properties from Continuously Cropped Soybean under Potted-Experiments

#### 2.4.1. Measurement of Biomass in Continuously Cropped Soybean under Potted-Experiments

**Figure 3:**
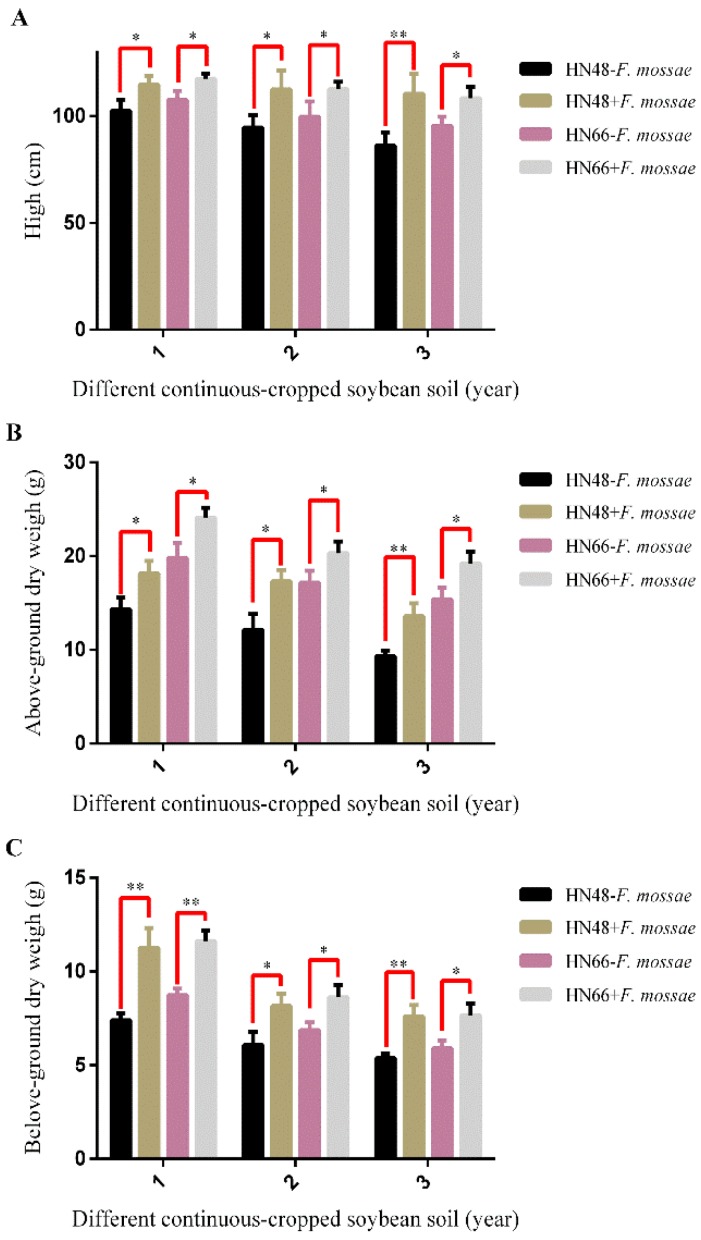
The biomass of two soybean cultivars under different continuous-cropped year soil in potted-experiments of soybean: 1 means soybean were planted the normal soil; 2 means soybean were planted in one-year continuous cropping soybean soil; and 3 means soybean were planted in three-year continuous cropping soybean soil. Each value represents the average of six independent experiments and the error bars represent standard deviations. Asterisks indicate the significance of differences between the samples. *p* values were calculated by Student’s *t*-test. Single asterisk indicates *p* < 0.05; double asterisks indicate *p* < 0.01.

**Figure 4:**
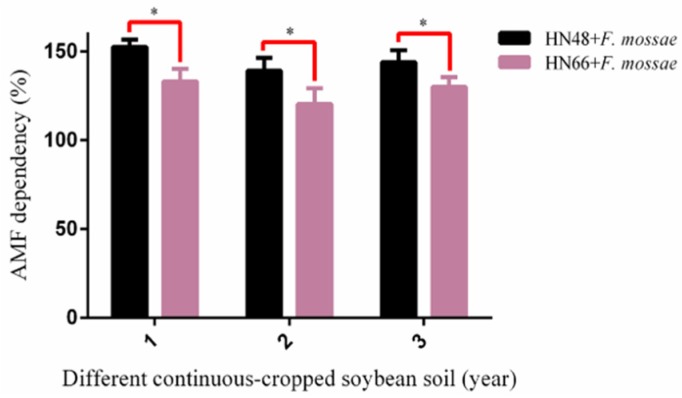
The AMF dependence of two soybean cultivars in the different year of continuously-cropped soybean soils: 1 means soybean were planted the normal soil; 2 means soybean were planted in one-year continuous cropping soybean soil; and 3 means soybean were planted in three-year continuous cropping soybean soil. Each value represents the average of six independent experiments and the error bars represent standard deviations. Asterisks indicate the significance of differences between the samples. *p* values were calculated by Student’s *t*-test. Single asterisk indicates *p* < 0.05.

With increasing years of continuous cropping, the plant height and above- and belowground dry weights declined from year to year in the two soybean cultivars (Figure 3), suggesting that the number of years of continuous cropping negatively affects soybean biomass. In addition, the relative effect of the year of continuous cropping on plant height, and above- and belowground dry weight differed depending on the soybean cultivar, suggesting that soybean cultivars with different genetic backgrounds can differ with respect to disease resistance. After *F. mosseae* inoculation, plant height, and above- and belowground dry weights in the two cultivars increased for all years of continuous cropping, suggesting that *F. mosseae* has a positive effect on plant biomass. The results of AMF dependence in the HN48 and HN66 showed that AMF dependence in HN48 was relatively high (Figure 4).

#### 2.4.2. Determination of Rhizosphere Soil Physicochemical Properties in Continuously Cropped Soybean

**Figure 5:**
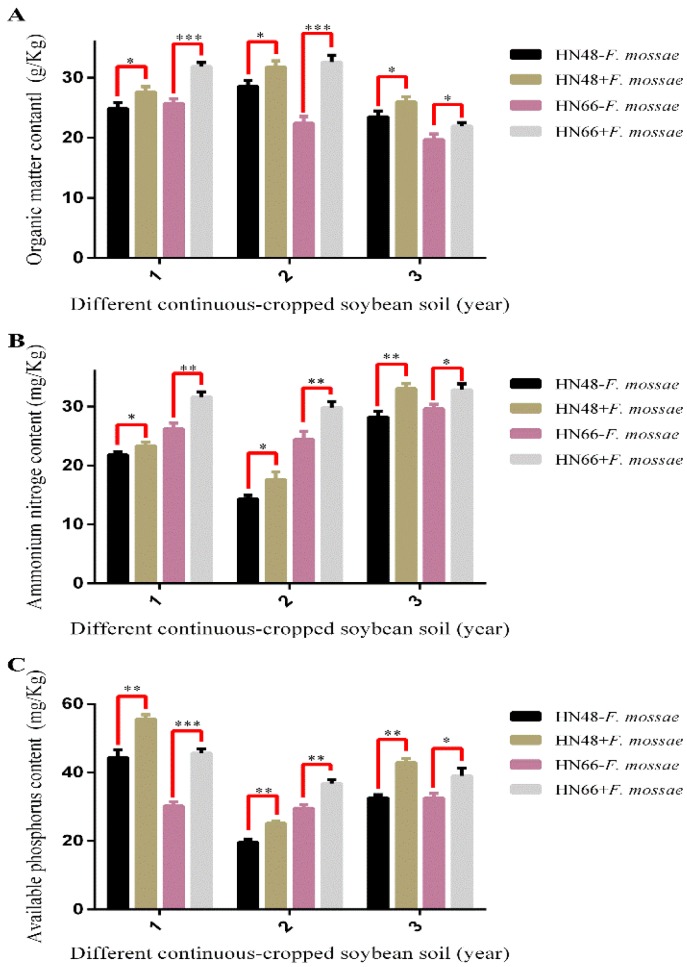
The physicochemical properties of two soybean cultivars rhizosphere soil under different continuous-cropped year soil in potted-experiments of soybean: 1 means soybean were planted the normal soil; 2 means soybean were planted in one-year continuous cropping soybean soil; and 3 means soybean were planted in three-year continuous cropping soybean soil. Each value represents the average of six independent experiments and the error bars represent standard deviations. Asterisks indicate the significance of differences between the samples. *p* values were calculated by Student’s *t*-test. Single asterisk indicates *p* < 0.05; double asterisks indicate *p* < 0.01; Triple asterisks indicate *p* < 0.001.

A soil physicochemical analyzer was used to determine the organic matter content, ammonia nitrogen content, and available phosphorus content of rhizosphere soil in the two soybean cultivars for the three years of continuous cropping (Figure 5).

With increasing years of continuous cropping, the organic matter content of rhizosphere soil gradually decreased in the HN66 without non-inoculated *F. mosseae* group (Figure 5A). HN48 without non-inoculated *F. mosseae* group showed the trend of first increasing and then decreasing (Figure 5A), suggesting that the organic matter content of rhizosphere soil is related to the continuous cropping year. After *F. mosseae* inoculation, the content of organic matter in rhizosphere soil for the two cultivars increased for all years of continuous cropping. With increasing years of continuous cropping, the amount of ammonia nitrogen in the rhizosphere soil showed the trend of first decreasing and then increasing (Figure 5B), suggesting a relationship between continuous cropping year and the ammonia nitrogen content of rhizosphere soil. After *F. mosseae* inoculation, the ammonia nitrogen contents of rhizosphere soil increased for the two cultivars over three years of continuous cropping (Figure 5B). With increasing years of continuous cropping, the available phosphorus contents of rhizosphere soil also showed the trend of first decreasing and then increasing (Figure 5C), suggesting a relationship between continuous cropping year and the available phosphorus content of rhizosphere soil. After *F. mosseae* inoculation, the available phosphorus contents of rhizosphere soil in both cultivars for the three years of continuous cropping all increased (Figure 5C).

## 3. Discussion

### 3.1. Effect of F. mosseae on The Disease Index of Root Rot in Two Soybean Cultivars over Three Years of Continuous Cropping Under Potted-Experiments

Long-term continuous cropping in soybean causes substantial changes to the microbial community structure in the rhizosphere soil as well as to the abundance and diversity of pathogenic fungi, such as *F. oxysporum* and *F. semitectum,* and the oomycete *Pythium* sp. [26]. With increasing years of continuous cropping, the soybean rhizosphere soil gradually transforms from “bacterial-type” high-fertility soil to “fungal-type” low-fertility soil, which eventually leads to a significantly higher incidence of soybean root rot under continuous cropping regimens [27,28]. In this study, morphological observation was used to determine the soybean root rot level in two cultivars during three years of continuous cropping under potted-experiments. Experimental results showed that, with increasing years of continuous cropping, the level of soybean root rot in the control group gradually increased from year to year. Therefore, the results of soybean root rot disease index indirectly showed that long-term continuous cropping in soybean can cause changes to the structure of the microbial community in rhizosphere soil.

AMF are mutualistic symbionts that interact with plant roots and microorganisms in the soil microecosystem. AMF can promote absorption of water and minerals (P, N, K, etc.) by plants and accelerate the formation of chloroplasts, improving photosynthetic rate and plant biomass [29,30,31]. AMF can also enhance tolerance to drought, salinity and alkalinity, and heavy metals by establishing a symbiotic relationship with the terrestrial tracheophyte root system. After *F. mosseae* inoculation, we found that the index of soybean root rot was significantly lower in the treatment group than in the control group, showing that inoculated *F. mosseae* could help soybean root uptake nutrients and enhance the soybean resist pathogenic fungi, ultimately alleviating the occurrence of soybean root rot under continuous cropping regimens [32,33]. We found that the control effect of *F. mosseae* on soybean root rot varied by soybean cultivar, suggesting that soybean genetic background plays a role in the effects mediated by *F. mosseae*. Therefore, our study verified that AMF can reduce the incidence of soybean root rot under continuous cropping regimens. The results of our study also showed that AMF can improve the rhizosphere microenvironment in continuously cropped soybean while maintaining stability of the soil microecosystem.

### 3.2. Effect of F. mosseae on Root Tissue Metabolites and Root Exudates Produced by the Two Cultivars over Three Years of Continuous Cropping under Potted-Experiments

Long-term continuous cropping in soybean causes substantial changes to the types and amounts of secondary metabolites secreted by the roots, leading to an enrichment of harmful secondary metabolites (phenolic acids, benzene, and esters), which eventually results in auto toxicity. We used GC-MS to determine the metabolites profiling of root tissue and root exudates from two soybean cultivars over three years of continuous cropping. Results showed that the metabolites profiling of both HN48 (root rot susceptible) and HN66 (root rot resistant) included hydrocarbons, esters, alcohols, benzene, and acids; the relative amounts of the metabolites differed, suggesting that the two soybean cultivars have different genetic backgrounds, leading to variations in resistance to biotic factors. To resist adverse effects, two types of allelochemicals (Bis(2-ethylhexyl) phthalate and dibutyl phthalate) are synthesized and secreted by soybean. The presence of allelochemicals in root exudates of continuously cropped soybean shows that the soybean root has become infected with a large number of pathogenic microorganisms, and, to resist adverse effects of pathogens, the roots synthesize and secrete these two allelochemicals that act against pathogenic microorganisms [16,34]. However, the secretion of allelochemicals can also inhibit the growth of the soybean plant itself. Therefore, our results show that the synthesis and secretion of metabolites in the soybean rhizosphere eventually causes a reduction in soybean biomass, yield, and quality [35,36].

After infecting soybean roots, AMF can increase the area and ability of the roots to absorb moisture and mineral elements through a massive mycelium structure. In regulating stress resistance in the host, AMF can play a role in the regulation of plant defensive proteins, defensive enzymes, and secondary metabolites, mainly by affecting the host-related metabolic pathways and defense gene expression. Our results showed that the differently abundant metabolites in HN48 and HN66 included hydrocarbons, acids, esters, benzene, and naphthalene, and that *F. mosseae* affects the synthesis and secretion of root exudates metabolites in continuously cropped soybean under potted-experiments. Comparisons of the types of differently abundant metabolites and the index of root rot between the control and treatment groups also suggested that *F. mosseae* can improve the rhizosphere microenvironment in continuously cropped soybean under potted-experiments [19,37,38]. Therefore, our study provides an experimental basis for further exploring the resistance of different soybean cultivars to infection by root rot pathogens and the way in which AMF can alleviate soybean root rot under potted-experiments.

### 3.3. Effect of F. mosseae on the Biomass and Physicochemical Properties of Rhizosphere Soil in Two Soybean Cultivars over Three Years of Continuous Cropping under Potted-Experiments

Long-term continuous cropping in soybean causes changes to the rhizosphere microbial community structure (from “bacterial type” high fertility soil to “fungal type” low fertility soil), enrichment of harmful secondary metabolites (phenolic acids, esters, and benzene), resulting in the changes in physicochemical properties of soybean rhizosphere soil (organic matter content and soil enzyme activity), which ultimately leads to reduction in soybean biomass and a higher incidence of root rot.

We measured the biomass, organic matter content, and available ammonium nitrogen and phosphorus contents of rhizosphere soil in two soybean cultivars over three years of continuous cropping under potted-experiments. Experimental results showed that with the increasing years of continuous cropping, soybean biomass gradually decreased in the control group. Additionally, continuous cropping affected the physicochemical properties of rhizosphere soil, thus reducing the ability of the soybean root to absorb enough nutrients. The poor nutritional status of soybean rhizosphere soil also inhibited the growth of the soybean root system, eventually resulting in the poor plant growth and a reduction in yield and quality. After *F. mosseae* inoculation, soybean biomass increased in the treatment group, showing that by infecting the soybean roots, *F. mosseae* can enhance the ability of soybean roots to absorb mineral nutrition and moisture through the formation of mycorrhizal structures such as arbuscules and vesicles [22,33,39]. We also found that the physicochemical indexes of rhizosphere soil were significantly higher than those in the control group, suggesting that *F. mosseae* can improve the rhizosphere microenvironment in continuously cropped soybean under potted-experiments, providing an experimental basis for applying AMF to reduce the incidence of soybean root rot in continuous cropping systems.

## 4. Materials and Methods

### 4.1. Soybean Cultivars and Experimental Microbial Agent

Two soybean cultivars with different root rot resistance were selected as the experimental material in this study: Hei-Nong 48 (susceptible to root rot, abbreviated HN48, purchased from Heilongjiang Academy of Agricultural Sciences, Harbin, China) and Hei-Nong 66 (resistant to root rot, abbreviated HN66, purchased from Heilongjiang Academy of Agricultural Sciences, Harbin, China). The fat content of HN48 is 18.43% and the protein content is 45.23%, while the fat and protein contents of HN66 are 21.25% and 37.68%, respectively.

The Arbuscular Mycorrhizal Fungus *Funneliformis mosseae* (CGMCC No. 3012), isolated from a continuously cropped soybean field in Heilongjiang Province, was chosen as the experimental AMF strain in this study. The inoculants used was a rhizosphere sand–soil mixture containing fungal spore and hyphens, obtained after using *Medicago sativa* as the host plant.

### 4.2. Growth and Maintenance of Soybean Plants

A pot experiment was used for soybean planting in this study. Each pot was filled with 4 kg air-dried soil which was sampled from the soybean rhizosphere at Year 0 of continuous cropping, Year 1 of continuous cropping and Year 3 of continuous cropping. The *F. mosseae* inoculant (45 g) was added to each pot, and thoroughly mixed into the soil. The control group was identical but without *F. mosseae* inoculation. Each treatment was repeated six times. After planting, pots of the same size, also sown with soybeans, were placed to surround the experimental pots as a protection barrier to prevent marginal effects.

### 4.3. Detection of Soybean Root Rot Index and AMF Colonization Rate

Starting thirty days after planting the soybeans, a root rot rating was performed every seven days [40]. Plants were scored for disease as follows: 0, no disease spots on the basal stem and axial root; 1, sporadic disease spots present; 2, flakey sporadic disease spots present; 3, diseased areas present on 25% of the root length; 4, diseased areas present on 33% of the root length and disease spots coalesce around the stem, but root not necrotic; and 5, diseased areas present on >50% of the root length.

In addition to root rot assays, the AMF colonization rate was also examined every seven days using acid fuchsin staining [41].

### 4.4. Test Sample Collection

When collecting soil samples, three soybean plants of the same cultivar and same treatment were randomly selected, and the 10–20 cm soil profile was removed with the soybean plant. The plant was then gently shaken, and the soil adhering to the soybean root surface in a layer 1–3 mm thick was collected as rhizosphere soil. The rhizosphere soil from roots of three soybean plants was collected with a brush and combined into a single sample. To sample the root tissue, the roots used for soil sample collection were washed, and roots from three randomly-selected plants were cut into pieces with scissors and combined into a single sample. Sampling was repeated six times. The root and soil samples from the different treatments in different years of continuous cropping were designated RC0, RC1, RC3, SC0, SC1, SC3 and RT0, RT1, RT3, ST0, ST1, and ST3. After collection, the samples were immediately taken to the laboratory, frozen in liquid nitrogen, and stored at −80 °C.

### 4.5. DNA Extraction and Specific Fragment Amplification

An established method using cetyl trimethyl ammonium bromide (CTAB) and the Omega genomic DNA E.Z.N.A. ^®^Soil DNA Kit [29] were used for total DNA extraction from the root and rhizosphere soil samples, respectively. DNA extracted from soybean roots and rhizosphere soil was stored at −20 °C prior to use in PCR assays. The primer pair NS1 (5′-GTAGTCATATGCTTGT CTC-3′) and FungGC (5′-CGCCCGCCGCGCCCCGCGCCCGGCCCGCCGCCCCCGCCCCATTCCCCG TTACCCGTTG-3′) [42] were used for the specific amplification of the fungal 18S rDNA (V1 + V2) region from the root and soil DNA samples. The PCR amplifications were performed as follows: pre-denaturation at 94 °C for 3 min, followed by 35 cycles of denaturation at 94 °C for 30 s, annealing at 57 °C for 1 min, and extension at 72 °C for 1 min, with a final extension at 72 °C for 10 min. Primers 5.25 (5′-ATCAACCTTTTGAGCTCG-3′) and NDL22 (5′-TGGTCCGTGTTTCAAGACG-3′) [43] were used to amplify the *F. mosseae* 18S rDNA NS31/Glol region. The PCR conditions were: pre-denaturation at 95 °C for 3 min, denaturation at 94 °C for 1 min, annealing at 58 °C for 1 min, extension at 72 °C for 1 min, for a total of 33 cycles, followed by a final extension at 72 °C for 5 min. After PCR amplification, the amplification products were examined by electrophoresis on a 1% agarose gel.

### 4.6. Extraction and Identification of Root Metabolites

Soybean roots (1.5 g) were ground to a powder using liquid nitrogen. Samples of 0.2 g root powder were extracted in 1 mL n-hexane in 2 mL centrifuge tubes for 24 h. After extraction, the tubes were centrifuged at 13,000 r/min for 10 min. The supernatant fluids were collected, filtered through a 0.22 µm membrane, and dried with nitrogen. For derivatization, 200 µL methoxyamine pyridine (15 mg/µL) was added, the samples were incubated at 70 °C for 1 h, after which 200 µL MSTFA (1% TMCS) solution was added and incubation at 70 °C was continued for a further 1 h. After the reaction, the derivatized solutions were cooled to room temperature for 30 min.

Accurately weighed samples of soybean rhizosphere soil (5 g) were ground in liquid nitrogen, transferred to 50 mL centrifuge tubes, and extracted for 24 h in 30 mL n-hexane. Samples were then centrifuged at 13,000 r/min for 10 min, and the supernatants filtered through a 0.22 µm membrane. After filtration, the supernatant liquid volume was reduced to 0.5 mL under vacuum, and a 200 µL sample was used in GC-MS detection.

GC-MS was used to analyze the metabolites in rhizosphere soil samples from the two cultivars in three years of continuous cropping. GC-MS detection parameters were as follows: injection port temperature of 250 °C; column temperature (using temperature programming) had an initial temperature of 50 °C, maintained for 5 min, increased to 150 °C at 10 °C/min, maintained for 5 min, increased to 250 °C at 5 °C/min, maintained for 10 min; the GC detector was a hydrogen flame ionization detector (FID); MS ion source was an electron impact ionization source (EI source); and the analyzer was a Quadrupole Mass Spectrometer.

### 4.7. Determination of Continuously Cropped Soybean Biomass and Rhizosphere Soil Physicochemical Properties

Oven-drying method was used to determine the above- and belowground dry weights of continuously cropped soybean. A calibrated scale was used to determine plant height, and a pH meter was used to determine the rhizosphere soil pH. The potassium dichromate method was used to determine the organic matter content of the soil. Ammonium nitrogen and the available phosphorus content in the soil were measured using a soil analyzer.

AMF dependency was expressed as the ratio of the dry weights of inoculated AMF plants and non-inoculated AMF plants.

### 4.8. Data Analysis

Microsoft Excel (Microsoft, Redmond, WA, USA) was used to record and for the initial processing of continuously cropped soybean biomass data, Prism 6 software (Version: 6.0, GraphPad Software, La Jolla, CA, USA) was used for *t*-test. R (version: 2.13, Mathsoft Inc., Cambridge, MA, USA) was for the GC-MS raw data processing, and SIMCA-p software (version 11.5, Umetrics, Umea, Sweden) was used to analyze the root exudates of continuously cropped soybean [44]. We used PLS-DA for the differential metabolite screening, and variable importance plot (VIP) values (VIP value > 1, and *p* < 0.05) were used to identify the differently abundant metabolites [24].

## 5. Conclusions

In this study, we aimed to study the effects of AMF inoculation in continuously cropped soybean under potted-experiments. We can draw the following conclusions:

- *F. mosseae* can reduce the incidence of root rot in continuously cropped soybean.
- *F. mosseae* inoculation can affect the metabolite profiling in soybean roots.
- Inoculation with *F. mosseae* increase biomass in continuously cropped soybean.
- *F. mosseae* inoculation can improve the soybean rhizosphere microenvironment.

## Figures and Tables

**Table 1:** The differently abundant metabolites from HN48 root tissue under different continuous-cropped year soil in potted-experiments.

Retention Time (min)	Compounds	Metabolic Level	VIP Value	*p* Value
11.049	Benzoic acid, 2-fluoro-, ethyl ester	Down	2.2149	0.0096
42.635	Bis(2-ethylhexyl) phthalate	Down	2.1669	0.0104
43.42	Hexacosane	Down	2.1321	0.0165
33.973	Hexadecanoic acid	Up	2.1125	0.0021
21.62	Dodecane	Up	2.0025	0.0214
43.13	Tricosane, 2-methyl-	Down	1.9423	0.0475
30.164	1,2-Benzenedic carboxylic acid, bis(2-methylpropyl) ester	Down	1.916	0.0235
31.87	n-Pentadecanoic acid	Down	1.9012	0.0032

**Table 2:** The differently abundant metabolites from HN66 root tissue under different continuous-cropped year soil in potted-experiments.

Retention Time (min)	Compounds	Metabolic Level	VIP Value	*p* Value
12.317	Propanoic acid, 2-(hydroxyl)-	Up	1.9188	0.0092
46.432	Tetracosane	Down	1.7592	0.0162
42.635	Bis(2-ethylhexyl) phthalate	Down	1.7348	0.0208
44.384	Heptadecane, 9-hexyl-	Down	1.7081	0.0024
33.943	Hexadecanoic acid	Up	1.6488	0.0196
31.803	Benzenepropanoic acid,3,5-bis(1,1-dimethylethyl)-4-hydroxy-, methyl ester	Down	1.6223	0.0157
37.588	*cis*-9-Hexadecenoic acid	Down	1.5762	0.0184

**Table 3:** The differently abundant metabolites from HN48 root exudates under different continuous-cropped year soil in potted-experiments.

Retention Time (min)	Compounds	Metabolic Level	VIP Value	*p* Value
12.218	Benzene, (1-methyl-1-butenyl)-	Down	2.1285	0.0452
39.282	Heptadecane, 2-methyl	Up	2.0193	0.0314
42.476	Bis(2-ethylhexyl) phthalate	Down	1.9912	0.0299
35.533	Heneicosane	Up	1.9749	0.0189
20.077	Phenol, 2,4-bis(1,1-dimethylethyl)	Down	1.8462	0.0329
19.953	Sulfurous acid, 2-propyl tetradecyl ester	Down	1.8423	0.0415
18.119	Naphthalene, 1,3-dimethyl-	Down	1.8116	0.0235
26.006	Heptadecane	Up	1.8056	0.0032

**Table 4:** The differently abundant metabolites from HN66 root exudates under different continuous-cropped year soil in potted-experiments.

Retention Time (min)	Compounds	Metabolic Level	VIP Value	*p* Value
42.476	Bis(2-ethylhexyl) phthalate	Down	2.1566	0.0252
34.814	Octacosane	Down	2.1169	0.0424
36.337	Octadecane, 2-methyl-	Up	1.9912	0.0299
31.742	Benzene, (1-methyl-1-butenyl)-	Down	1.9749	0.0189
32.181	Dibutyl phthalate	Down	1.9156	0.0154
44.694	Tricosane	Up	1.8641	0.0077
46.108	Tetracosane	Up	1.8544	0.0044
20.077	Phenol, 2,4-bis(1,1-dimethylethyl)	Down	1.8356	0.0012
38.422	Octadecane	Up	1.638	0.0216
26.03	Heptadecane	Down	1.638	0.0216

## Supplementary Materials

The following are available online at http://www.mdpi.com/1422-0067/19/8/2160/s1, Figure S1: The total DNA PCR amplification results of root samples (left) and soil samples (right) in the different continuously-cropped year soil and different soybean cultivars (HN48 and HN66), Figure S2: The fungal 18S rRNA (V1 + V2) PCR amplification results of root samples (left) and soil samples (right) in the different continuously-cropped year soil and different soybean cultivars (HN48 and HN66), Figure S3: The *F. mosseae* 18S rRNA NS31/Glol PCR amplification results of HN48 (A) and HN66 (B) root samples in the different continuously-cropped year soil, Figure S4: Compared the root tissue metabolome of non-inoculated *F. mosseae* to inoculated *F. mosseae* in HN48 under different year of soybean continuously-cropped soils by potted-experiments, Figure S5: Compared the root tissue metabolome of non-inoculated *F. mosseae* to inoculated *F. mosseae* in HN66 under different year of soybean continuously-cropped soils by potted-experiments, Figure S6: Compared the root exudates of non-inoculated *F. mosseae* to inoculated *F. mosseae* in HN48 under different year of soybean continuously-cropped soils by potted-experiments, Figure S7: Compared the root exudates of non-inoculated *F. mosseae* to inoculated *F. mosseae* in HN66 under different year of soybean continuously-cropped soils by potted-experiments, Table S1: The relative abundance (%) of differentially metabolites from HN48 root tissue under different continuous-cropped year soil in potted-experiments, Table S2: The relative abundance (%) of differentially metabolites from HN66 root tissue under different continuous-cropped year soil in potted-experiments, Table S3: The relative abundance (%) of differentially metabolites from HN48 root exudates under different continuous-cropped year soil in potted-experiments, Table S4: The relative abundance (%) of differentially metabolites from HN66 root exudates under different continuous-cropped year soil in potted-experiments.
