# Supplementary material for: Effects of Funneliformis mosseae on Root Metabolites and Rhizosphere Soil Properties to Continuously-Cropped Soybean in the Potted-Experiments
Source: Int J Mol Sci. 2018 Jul 24;19(8):2160. doi: 10.3390/ijms19082160 (PMC6121952; doi:10.3390/ijms19082160)
Supplement: Supplementary file 1 [file ijms-19-02160-s001.pdf]

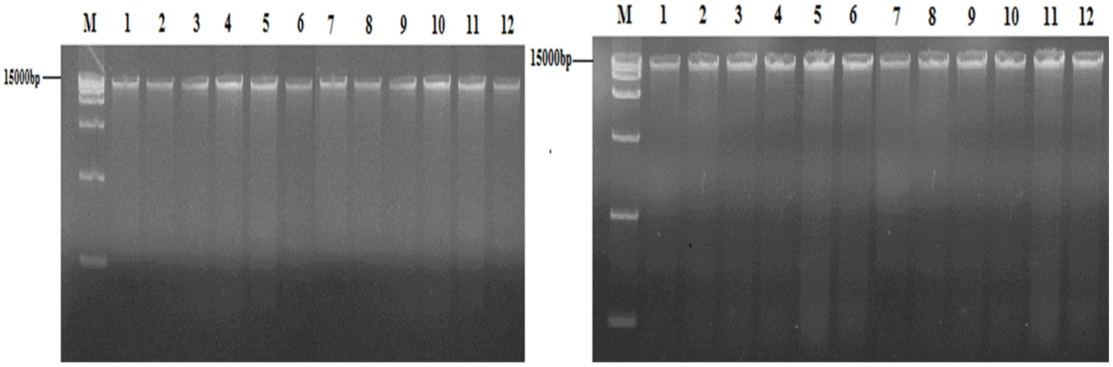

Figure S1. The total DNA PCR amplification results of root samples (left) and soil samples (right) in the different continuously-cropped year soil and different soybean cultivars (HN48 and HN66). M represented DL15000 Marker; 1-3 represented the total DNA PCR amplification results of HN48 roots/soils sample in the normal soil, 1 year continuous cropping soybean soil, 3 year continuous cropping soybean soil (control group, non-inoculated *F. mosseae*), respectively; 4-6 represented the total DNA PCR amplification results of HN48 roots/soils samples (the treatment group) in the normal soil, 1 year continuous cropping soybean soil, 3 year continuous cropping soybean soil (treatment group, inoculated *F. mosseae*), respectively; 7-9 represented the total DNA PCR amplification results of HN66 roots/soils samples (the control group) in the normal soil, 1 year continuous cropping soybean soil, 3 year continuous cropping soybean soil (control group, non-inoculated *F. mosseae*), respectively; 10-12 represented the total DNA PCR amplification results of HN66 roots/soils samples (the treatment group) in the normal soil, 1 year continuous cropping soybean soil, 3 year continuous cropping soybean soil (treatment group, inoculated *F. mosseae*), respectively.

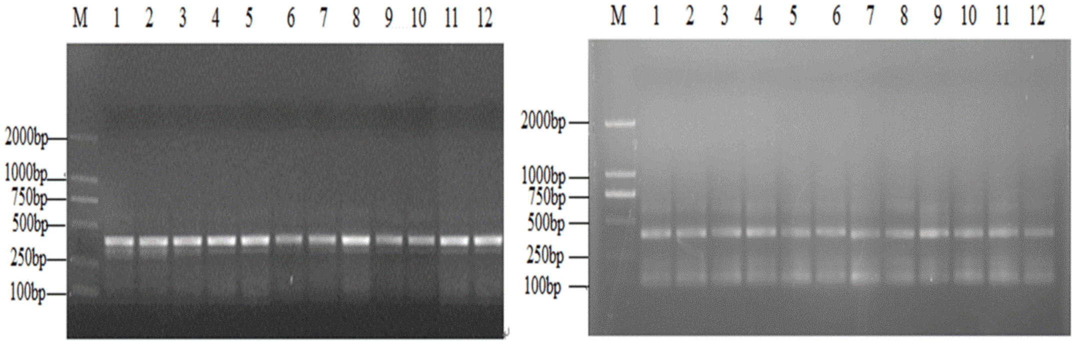

Figure S2. The fungal 18S rRNA（V1+V2）PCR amplification results of root samples (left) and soil samples (right) in the different continuously-cropped year soil and different soybean cultivars (HN48 and HN66). M represented DL15000 Marker; 1-3 represented the fungal 18S rRNA（V1+V2）PCR amplification results of HN48 roots/soils sample in the normal soil, 1 year continuous cropping soybean soil, 3 year continuous cropping soybean soil (control group, non-inoculated *F. mosseae*), respectively; 4-6 represented the fungal 18S rRNA（V1+V2）PCR amplification results of HN48 roots/soils samples in the normal soil, 1 year continuous cropping soybean soil, 3 year continuous cropping soybean soil (treatment group, inoculated *F. mosseae*), respectively; 7-9 represented the fungal 18S rRNA（V1+V2）PCR amplification results of HN66 roots/soils samples in the normal soil, 1 year continuous cropping soybean soil, 3 year continuous cropping soybean soil (control group, non-inoculated *F. mosseae*), respectively; 10-12 represented the fungal 18S rRNA（V1+V2）PCR amplification results of HN66 roots/soils samples in the normal soil, 1 year continuous cropping soybean soil, 3 year continuous cropping soybean soil (treatment group, inoculated *F. mosseae*), respectively;.

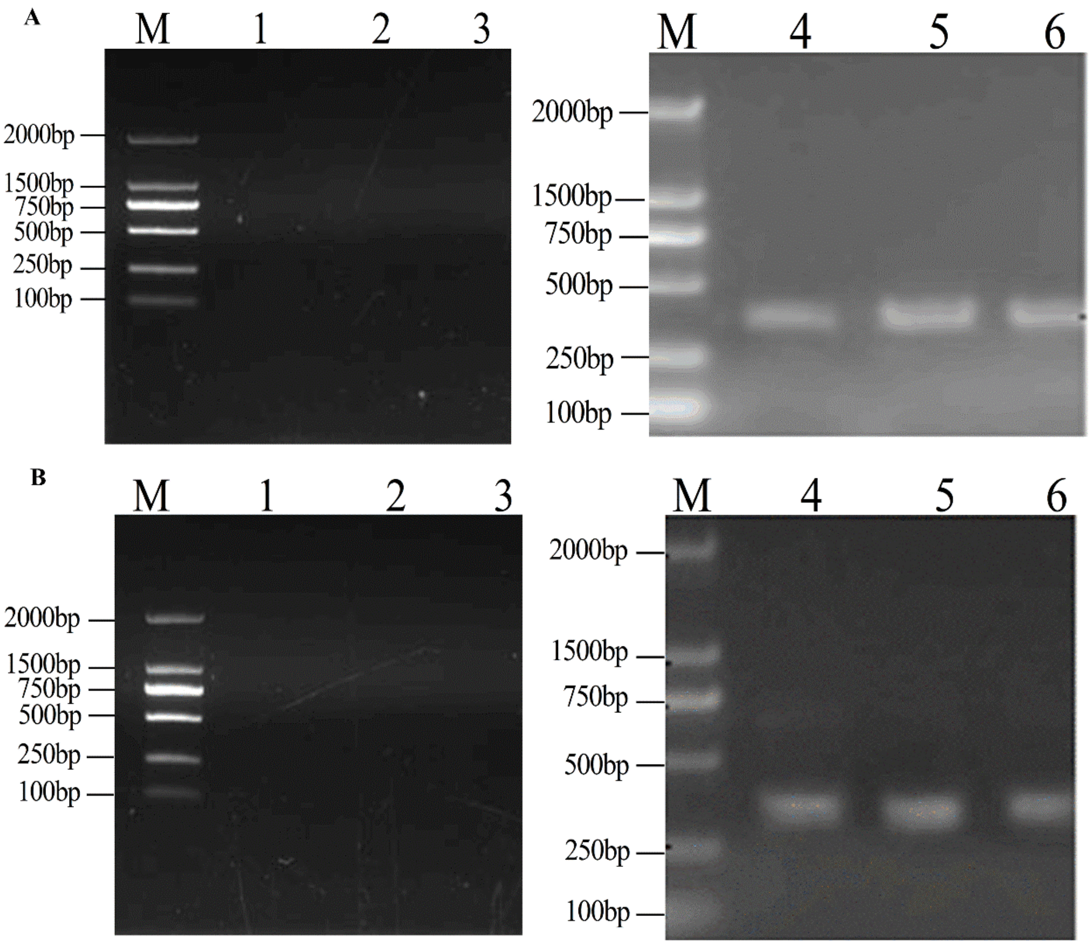

Figure S3. The *F. mosseae* 18S rRNA NS31/Glol PCR amplification results of HN48 (A) and HN66 (B) root samples in the different continuously-cropped year soil. M represented DL15000 Marker; 1-3 represented the *F. mosseae* 18S rRNA NS31/Glol PCR amplification results of HN48/HN66 roots sample in the normal soil, 1 year continuous cropping soybean soil, 3 year continuous cropping soybean soil (control group, non-inoculated *F. mosseae*), respectively; 4-6 represented the *F. mosseae* 18S rRNA NS31/Glol PCR amplification results of HN48/HN66 roots sample in the normal soil, 1 year continuous cropping soybean soil, 3 year continuous cropping soybean soil (treatment group, inoculated *F. mosseae*), respectively.

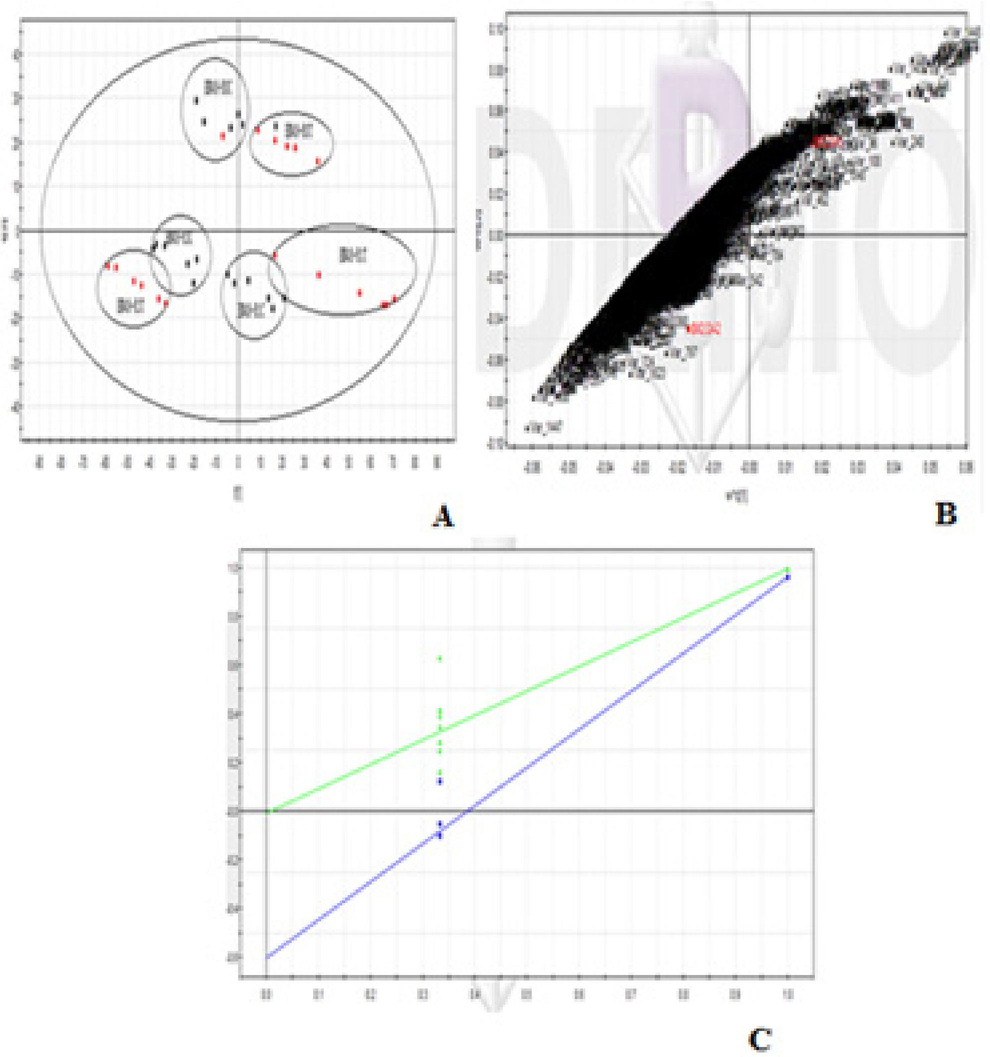

Figure S4. Compared the root tissue metabolome of non-inoculated *F. mosseae* to inoculated *F. mosseae* in HN48 under different year of soybean continuously-cropped soils by potted-experiments. A represents principal component analysis result; B represents partial least-squares analysis result; C represents model validation result. The parameters of this model were as follows: A=2, R2X=0.922, R2Y=0.942, Q2Y=0.883, R2-intercept=0.224 (<0.4), Q2-intercept=-0.023 (<0.05). A is the number of principal components selected by this model, R2X and R2Y represent the explanatory ability to the treatment data of this model, Q2Y represents the predictive ability of this model to treatment design, and R2 and the Q2-intercept further verified whether the treatment design model is overfitting. This treatment model had some predictive ability and stability. In the PLS-DA loading diagram, the farther the distance of the metabolites to the center spot, the greater their contribution to group separation.

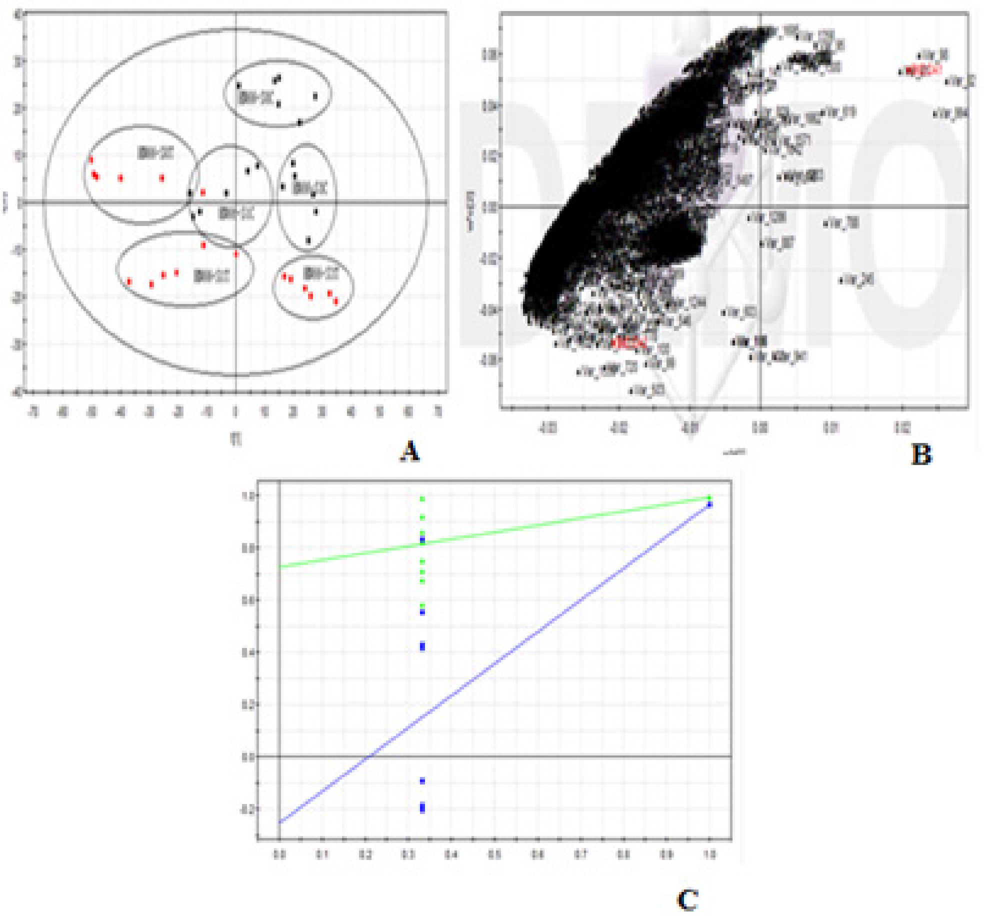

Figure S5. Compared the root tissue metabolome of non-inoculated *F. mosseae* to inoculated *F. mosseae* in HN66 under different year of soybean continuously-cropped soils by potted-experiments. A represents principal component analysis result; B represents partial least-squares analysis result; C represents model validation result. The parameters of this model were as follows: A=2, R2X=0.942, R2Y=0.983, Q2Y=0.965, R2-intercept=0.217 (<0.4), Q2-intercept=-0.035 (<0.05), A is the number of principal components selected by this model, R2X and R2Y represent the explanatory ability to the treatment data of this model, Q2Y represents the predictive ability of this model to treatment design, and R2 and the Q2-intercept further verified whether the treatment design model is overfitting. This treatment model had some predictive ability and stability. In the PLS-DA loading diagram, the farther the distance of metabolites from the center spot, the greater the contribution to group separation; a total of seven chromatographic peaks were screened out in combination with the VIP value (generally VIP > 1).

Table S1. The relative abundance (%) of differentially metabolites from HN48 root tissue under different continuous-cropped year soil in potted-experiments.

Compounds	0		1		3	
	C	T	C	T	C	T
Benzoic acid, 2-fluoro-, ethyl ester	0.0544 ±0.0021	0.0381 ±0.0019	0.0851 ±0.0051	0.0717 ±0.0036	0.0517 ±0.0026	0.0367 ±0.0017
Bis(2-ethylhexyl) phthalate	0.0433 ±0.0016	0.0311 ±0.0016	0.0597 ± 0.0036	0.0372 ±0.0019	0.0651 ±0.0033	0.0487 ±0.0024
Hexacosane	0.0311 ±0.0011	0.0219 ±0.0011	0.0402 ±0.0024	0.0264 ±0.0013	0.0307 ±0.0014	0.0175 0.0007
Hexadecanoic acid	0.0481	0.0605	0.0256	0.0374	0.0139 0.0007	0.0206 0.0011

	±0.0018	±0.0031	±0.0015	±0.0019		
Dodecane	0.0124 ±0.0004	0.0244 ±0.0012	0.0171 ±0.0010	0.0241 ±0.0012	0.0102 ±0.0006	0.0185 0.0008
Tricosane, 2-methyl-	0.0191 ±0.0008	0.0112 ±0.0003	0.0291 ±0.0017	0.0157 ±0.0008	0.0142 0.0007	0.0074 0.0004
1,2-Benzenedic carboxylic acid, bis(2-methylpropyl) ester	0.0542 ±0.0022	0.0411 ±0.0021	0.0252 ±0.0015	0.0122 ±0.0006	0.0187 0.0010	0.0102 0.0004
n-Pentadecanoic acid	0.0414 ±0.0016	0.0302 ±0.0015	0.0751 ±0.0045	0.0647 ±0.0033	0.0472 0.0024	0.0314 0.0016

Note; 0 means soybean were planted the normal soil; 1 means soybean were planted 1 year continuous cropping soybean soil; 3 means soybean were planted 3 year continuous cropping soybean soil; C means non-inoculated *F. mosseae*; T means inoculated *F. mosseae*.

Table S2. The relative abundance (%) of differentially metabolites from HN66 root tissue under different continuous-cropped year soil in potted-experiments.

Compounds	0		1		3	
	C	T	C	T	C	T
Propanoic acid, 2-(hydroxyl)-	0.0114 ±0.0006	0.0321 ±0.0016	0.0208 ±0.0010	0.0412 ±0.0021	0.0082 ±0.0004	0.0322 ±0.0016
Tetracosane	0.0261 ±0.0014	0.017 ±0.0009	0.0358 ±0.0018	0.0121 ±0.0007	0.0263 ±0.0013	0.0074 ±0.0004
Bis(2-ethylhexyl) phthalate	0.0641 ±0.0032	0.0332 ±0.0017	0.0439 ±0.0022	0.0259 ±0.0013	0.0491 ±0.0025	0.0344 ±0.0017
Heptadecane, 9-hexyl-	0.0274 ±0.0014	0.0138 ±0.0007	0.0175 ±0.0009	0.0086 ±0.0004	0.0253 ±0.0013	0.0156 ±0.0008
Hexadecanoic acid	0.082 ±0.0043	0.0225 ±0.0011	0.0142 0.0007	0.0388 ±0.0020	0.0127 ±0.0006	0.0342 ±0.0016
Benzenepropanoic acid,3,5-bis(1,1-dimethylethyl)-4-hydroxy-, methyl ester	0.0823 ±0.0042	0.0625 ±0.0032	0.0556 ±0.0028	0.0371 ±0.0018	0.0308 ±0.0014	0.0114 ±0.0006
cis-9-Hexadecenoic acid	0.0522 ±0.0027	0.0387 ±0.0018	0.0296 ±0.0011	0.0129 ±0.0007	0.0325 ±0.0015	0.0181 ±0.0011

Note; 0 means soybean were planted the normal soil; 1 means soybean were planted 1 year continuous cropping soybean soil; 3 means soybean were planted 3 year continuous cropping soybean soil; C means non-inoculated *F. mosseae*; T means inoculated *F. mosseae*.

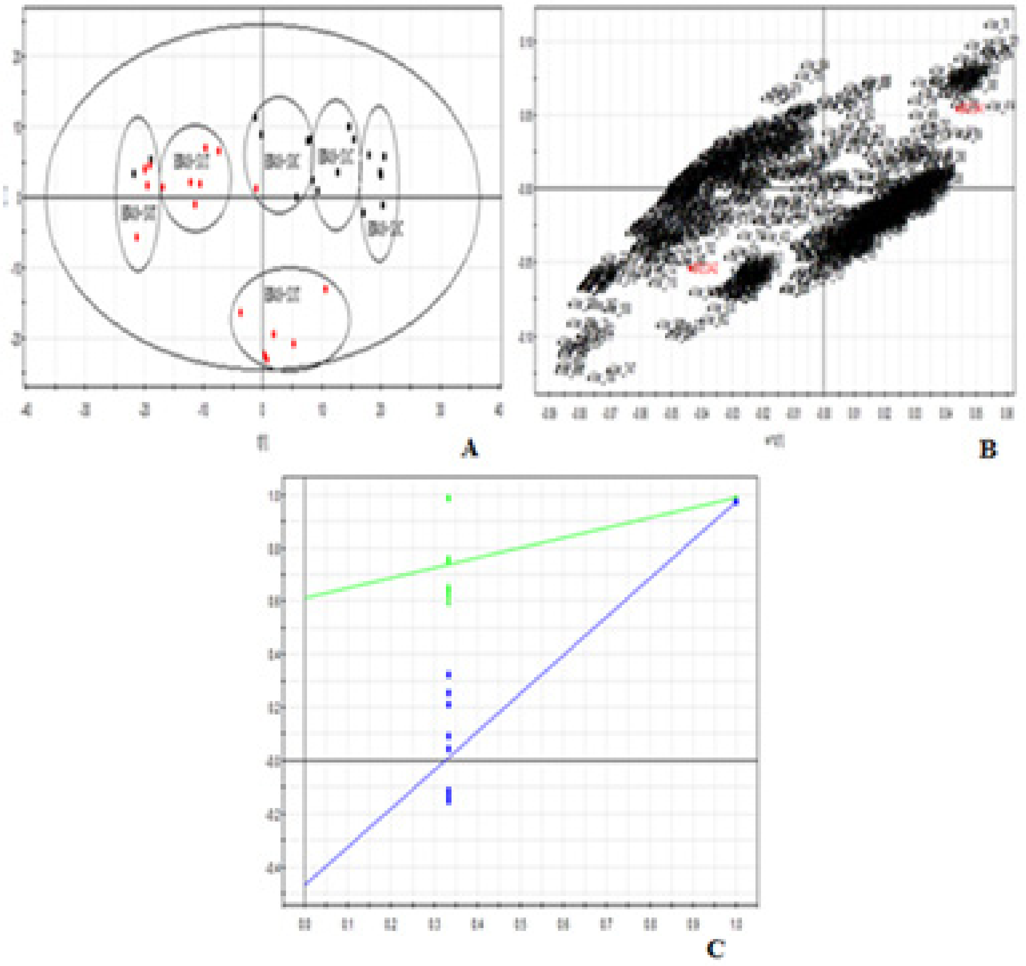

Figure. S6. Compared the root exudates of non-inoculated *F. mosseae* to inoculated *F. mosseae* in HN48 under different year of soybean continuously-cropped soils by potted-experiments. A represents principal component analysis result; B represents partial least-squares analysis result; C represents model validation result. The parameters of this model were as follows: A=2, R2X=0.903, R2Y=0.961, Q2Y=0.829, R2-intercept=0.204 (<0.4), Q2-intercept=-0.027 (<0.05), A is the number of principal components selected by this model, R2X and R2Y represent the explanatory ability to the treatment data of this model, Q2Y represents the predictive ability of this model to treatment design, and R2 and the Q2-intercept further verified whether the treatment design model is overfitting. This treatment model had some predictive ability and stability. In the PLS-DA loading diagram, the farther the distance of the metabolites from the center spot, the greater the contribution to group separation; a total of eight chromatographic peaks were screened out in combination with VIP values (generally VIP > 1).

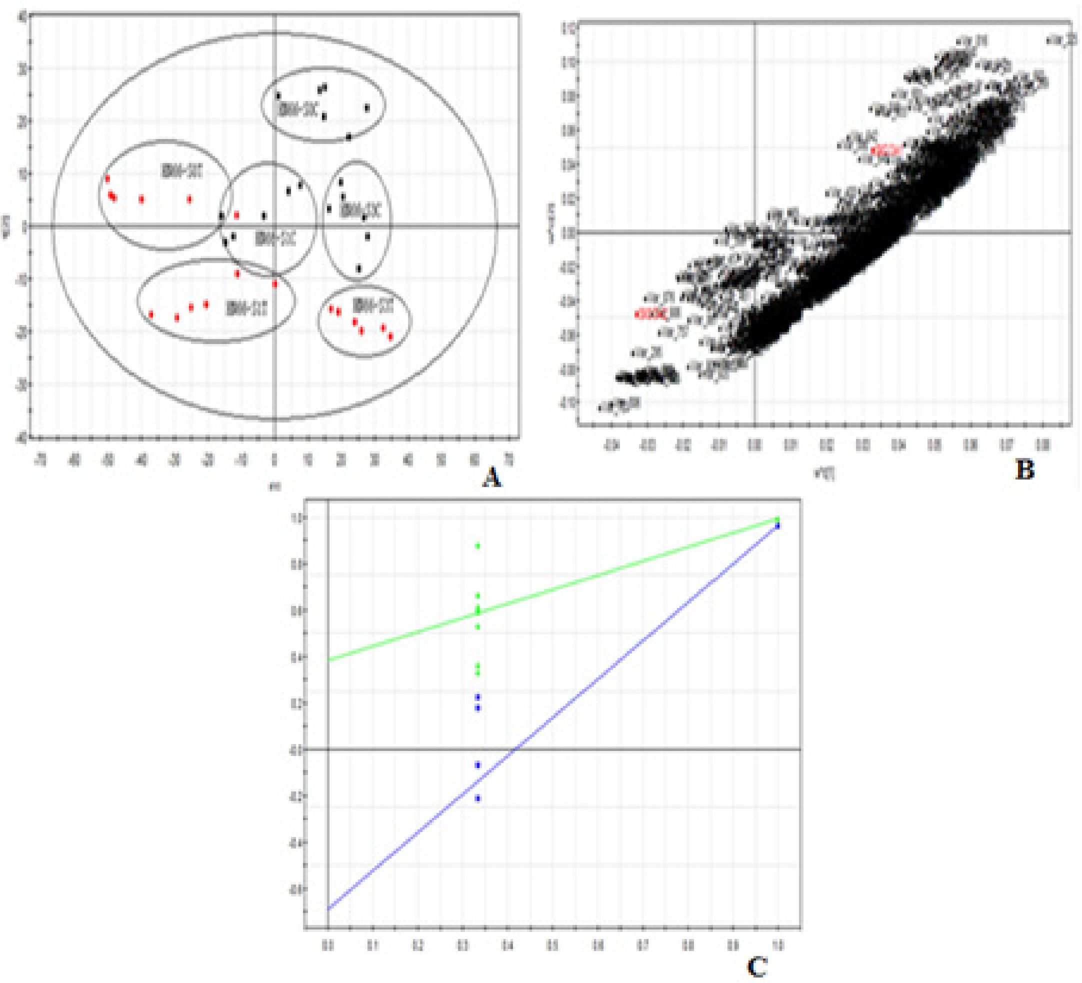

Figure S7. Compared the root exudates of non-inoculated *F. mosseae* to inoculated *F. mosseae* in HN66 under different year of soybean continuously-cropped soils by potted-experiments. A represents principal component analysis result; B represents partial least-squares analysis result; C represents model validation result the parameters of this model were as follows: A=2, R2X=0.913, R2Y=0.949, Q2Y=0.829, R2-intercept=0.204 (<0.4), Q2-intercept=-0.027 (<0.05). A is the number of principal components selected by this model, and R2X and R2Y represent the explanatory ability to the treatment data of this model; the treatment model had some predictive ability and stability. In the PLS-DA loading diagram, the farther the distance of the metabolites from the center, the greater the contribution to group separation; 10 chromatographic peaks were screened out in combination with their VIP values (generally VIP>1).

Table S3. The relative abundance (%) of differentially metabolites from HN48 root exudates under different continuous-cropped year soil in potted-experiments.

Compounds	0		1		3	
	C	T	C	T	C	T
Benzene, (1-methyl-1-butenyl)-	0.0332 ±0.0017	0.0103 ±0.0004	0.0417 ±0.0021	0.0161 ±0.0007	0.0331 ±0.0014	0.0154 ±0.0007
Heptadecane, 2-methyl	0.0283 ±0.0014	0.0366 ±0.0015	0.0159 ±0.0008	0.0397 ±0.0018	0.0171 ±0.0007	0.0364 ±0.0016
Bis(2-ethylhexyl) phthalate	0.0149 ±0.0007	0.0051 ±0.0002	0.0262 ±0.0013	0.0138 ±0.0006	0.0441 ±0.0018	0.0171 ±0.0007
Heneicosane	0.0194 ±0.0010	0.0376 ±0.0015	0.0142 ±0.0007	0.0294 ±.0013	0.0174 ±0.0007	0.0316 ±0.0014
Phenol, 2,4-bis(1,1-dimethylethyl)	0.0272 ±0.0014	0.0108 ±0.0004	0.0192 ±0.0009	0.0074 ±0.0003	0.0281 ±0.0011	0.0113 ±0.0005
Sulfurous acid, 2-propyl tetradecyl ester	0.0241 ±0.0012	0.0143 ±0.0006	0.0329 ±0.0016	0.0201 ±0.0011	0.0234 ±0.0009	0.0097 ±0.0004
Naphthalene, 1,3-dimethyl-	0.0175 ±0.0009	0.097 ±0.0039	0.0271 ±0.0013	0.0145 ±0.0007	0.0152 ±0.0006	0.0072 ±0.0003
Heptadecane	0.0172 ±0.0008	0.0291 ±0.0012	0.0241 0.0012±	0.0374 ±0.0017	0.0212 ±0.0009	0.0322 ±0.0014

Note; 0 means soybean were planted the normal soil; 1 means soybean were planted 1 year continuous cropping soybean soil; 3 means soybean were planted 3 year continuous cropping soybean soil; C means non-inoculated *F. mosseae*; T means inoculated *F. mosseae*.

Table S4. The relative abundance (%) of differentially metabolites from HN66 root exudates under different continuous-cropped year soil in potted-experiments.

Compounds	0		1		3	
	C	T	C	T	C	T
Bis(2-ethylhexyl) phthalate	0.0181 ±0.0008	0.0067 ±0.0004	0.0579 ±0.0029	0.0208 ±0.0010	0.0688 ±0.0029	0.0374 ±0.0019
Octacosane	0.0102 ±0.0004	0.0043 ±0.0002	0.0359 ±0.0018	0.0195 ±0.0009	0.0413 ±0.0017	0.0286 ±0.0015
Octadecane, 2-methyl-	0.0093 ±0.0004	0.0176 ±0.0009	0.0177 ±0.0009	0.0265 ±0.0013	0.0167 ±0.0007	0.0347 ±0.0018
Benzene, (1-methyl-1-butenyl)-	0.0214 ±0.0009	0.0167 ±0.0008	0.0327 ±0.0016	0.0145 ±0.0007	0.0226 ±0.0009	0.0137 ±0.0007
Dibutyl phthalate	0.0145 ±0.0006	0.0071 ±0.0004	0.0241 ±0.0012	0.0142 ±0.0019	0.0354 ±0.0015	0.0162 ±0.0008
Tricosane	0.0338 ±0.0014	0.0415 ±0.0021	0.0266 ±0.0013	0.0397 ±0.0020	0.0396 ±0.0017	0.0559 ±0.0029
Tetracosane	0.0332 ±0.0013	0.0433 ±0.0021	0.0318 ±0.0016	0.0425 ±0.0008	0.0258 ±0.0011	0.0362 ±0.0018
Phenol, 2,4-bis(1,1-dimethylethyl)	0.0172 0.0007	0.0098 ±0.0005	0.0241 ±0.0012	0.0158 ±0.0015	0.0359 ±0.0015	0.0232 ±0.0012
Octadecane	0.0185 ±0.0008	0.0243 ±0.0012	0.022 ±0.0011	0.0308 ±0.0015	0.0337 ±0.0014	0.0407 ±0.0021
Heptadecane	0.0277 ±0.0012	0.0215 ±0.0011	0.0365 ±0.0018	0.0288 ±0.0014	0.0275 ±0.0012	0.0204 ±0.0010

Note; 0 means soybean were planted the normal soil; 1 means soybean were planted 1 year continuous cropping soybean soil; 3 means soybean were planted 3 year continuous cropping soybean soil; C means non-inoculated *F. mosseae*; T means inoculated *F. mosseae*.
